# Tunable continuous wave emission via phase-matched second harmonic generation in a ZnSe microcylindrical resonator

**DOI:** 10.1038/srep11798

**Published:** 2015-07-02

**Authors:** N. Vukovic, N. Healy, J. R. Sparks, J. V. Badding, P. Horak, A. C. Peacock

**Affiliations:** 1 Optoelectronics Research Centre, University of Southampton, Southampton SO17 1BJ, UK; 2 Department of Chemistry and Materials Research Institute, Pennsylvania State University, 16802 PA, USA

## Abstract

Whispering gallery mode microresonators made from crystalline materials are of great interest for studies of low threshold nonlinear phenomena. Compared to amorphous materials, crystalline structures often exhibit desirable properties such as high indices of refraction, high nonlinearities, and large windows of transparency, making them ideal for use in frequency comb generation, microlasing and all-optical processing. In particular, crystalline materials can also possess a non-centrosymmetric structure which gives rise to the second order nonlinearity, necessary for three photon processes such as frequency doubling and parametric down-conversion. Here we report a novel route to fabricating crystalline zinc selenide microcylindrical resonators from our semiconductor fibre platform and demonstrate their use for tunable, low power continuous wave second harmonic generation. Visible red light is observed when pumped with a telecommunications band source by a process that is phase-matched between different higher order radial modes, possible due to the good spatial overlap between the pump and signal in the small volume resonator. By exploiting the geometrical flexibility offered by the fibre platform together with the ultra-wide 500–22000 nm transmission window of the ZnSe material, we expect these resonators to find use in applications ranging from spectroscopy to quantum information systems.

Microresonators that strongly confine light both spatially and temporally hold great potential for the construction of compact, low power devices. When made from highly nonlinear materials, these resonators are ideal for studying light matter interactions and photon dynamics from both a classical and quantum optical perspective. To date, the majority of nonlinear microresonators have been fabricated from amorphous materials that are molded under surface tension reshaping to have ultra-smooth surfaces^1^,^2^,^3^. This has allowed for a number of important demonstrations of processes that are governed by the third order susceptibility *χ*^(3)^, including broadband wavelength conversion via third harmonic generation (THG)^4^, four-wave mixing based oscillators^5^ and frequency combs^6^, as well as Raman lasing^7^. More recently there has been increased interest in fabricating resonators from crystalline materials owing to their high nonlinearities and broad transparency windows^8^,^9^,^10^. Specifically, materials that are non-centrosymmetric, and thus possess the larger second order susceptibility *χ*^(2)^, are highly desirable for ultra-broadband wavelength conversion and amplification extending from the visible to the mid-infrared regimes. However, in general it is more challenging to shape crystalline materials into a smooth form and typically such resonators are fabricated via mechanical polishing^9^ or chemical etching^11^.

Many of the initial demonstrations of nonlinear processing in crystalline microresonators have made use of silicon platforms, where it is possible to leverage the advanced nano-fabrication techniques to fashion disk and ring resonators^8^,^11^. However, more recently this work has been extended to compound semiconductor materials such as AlN^12^,^13^, GaAs^14^ and AlGaAs^15^, to access the *χ*^(2)^ nonlinearity both for nonlinear frequency conversion and electro-optic modulation. Typically these planar-based semiconductor resonators are defined using standard photolithography and etching methods, resulting in surface roughness levels on the order of a few nanometers. Owing to the high material index of the resonators, this degree of roughness can significantly limit the highest obtainable quality factors *Q* within the micron-sized structures preferred for low threshold nonlinear applications^11^. In an alternative approach, we have developed a method to fabricate microcylindrical resonators starting from our semiconductor fibre platform that have ultra-smooth surfaces defined by the silica capillary into which the material is deposited^16^. By exploiting the small mode volumes and low scattering losses of resonators fabricated from silicon core fibres, we have recently demonstrated low power, ultra-fast Kerr modulation and switching^17^.

In this paper, we present characterizations of the first compound semiconductor fibre-based resonators and demonstrate their use for low threshold, continuous emission of red light via second harmonic generation (SHG). The resonators are fabricated from ZnSe core optical fibres, which have been shown to have losses ~1 dB/cm across most of the near-infrared wavelength region^18^. Compared to the micro-disk resonators, our cylindrical platform offers an additional degree of freedom through the unconstrained height of the whispering gallery modes (WGMs), which allows for continuous tuning of the coupled mode properties to satisfy phase-matching under various conditions. Importantly, although SHG has been demonstrated in ZnSe structures using quasi-phase-matching type processes^19^,^20^, this is the first time that modal phase-matching in a resonant ZnSe cavity has been employed. The flexibility of the fibre platform opens up new routes to developing continuously tunable sources that could be phase-matched across large regions of the broad transmission window of the material.

## Results

### Resonator characterization

**Figure 1:**
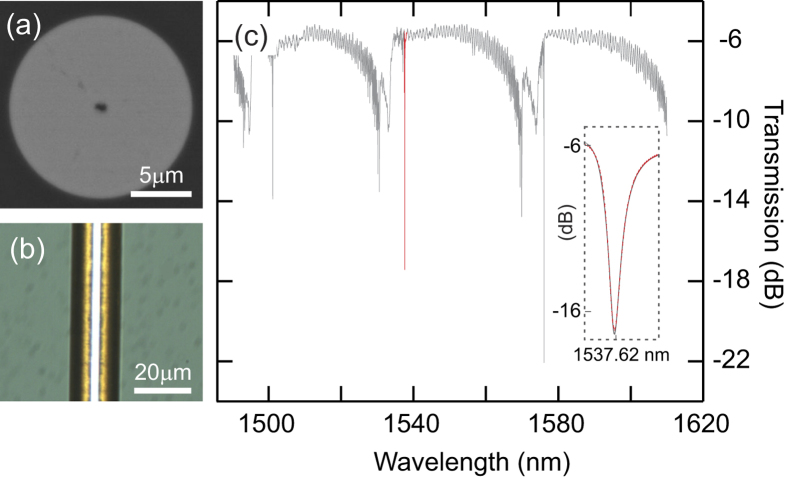
ZnSe resonator characterization. (**a**) SEM of ~15 *μ*m ZnSe core fibre. (**b**) Microscope image of etched ZnSe core to form the microresonator. (**c**) Typical transmission spectrum for a ~15 *μ*m diameter resonator; inset shows a close up of the resonance at *λ* ~ 1537 nm fitted with a fitted Lorentzian line shape (dashed red curve).

We start by characterizing a microcylindrical resonator that was fabricated from a ZnSe fibre with a ~15 *μ*m core diameter (see Methods). Before the resonator is formed, the optical loss of the core material was estimated via a cutback method to be 0.9 dB/cm at 1550 nm and 3.3 dB/cm at 800 nm. We note that some of this transmission loss can be attributed to a small <500 nm hole that remains in the centre of the fibre core following the deposition (see Methods), evident in the scanning electron microscope (SEM) image of the fibre core in Fig. 1(a). Thus we expect the actual ZnSe material loss to be slightly lower, especially at the shorter wavelengths. Furthermore, although the material is deposited in a polycrystalline form^18^, the measured losses are approaching some of the lowest values obtained in other popular polycrystalline *χ*^(2)^ materials such as AlN^12^. Following the transmission measurements, the microcylindrical resonator was revealed by simply etching the silica template away from the ZnSe core, as shown in Fig. 1(b). As the deposited material conforms to the ultra-smooth surface of the capillary template^21^, this ultimately defines the surface roughness of the resonator. The high quality of the etched core has been verified via a ZeScope 3D optical profiler, from which we obtained a root-mean-square roughness of ~0.2 nm^22^.

A standard tapered silica fibre coupling approach was used to measure the transmission spectrum of the microcylindrical resonator over the wavelength range 1490–1610 nm, as shown in Fig. 1(c) (see Methods). This spectrum clearly shows the sharp resonances associated with coupling to localized WGMs, with extinction ratios up to 16 dB. The loaded quality factors were estimated by fitting the resonances with Lorentzian line shapes, and are found to be of the order of *Q*_*l*_ = *λ*_*r*_/Δ*λ*_FWHM_ ~ 10^4^, with a maximum value of *Q*_*l*_ ~ 5 × 10^4^ for the resonance at *λ*_*r*_ = 1537.6 nm (see inset of Fig. 1(c)). Owing to the small dimensions of the high index resonator, the free spectral range (FSR) is relatively large ~38 nm, limiting the number of modes available for phase-matching SHG. However, as the generated harmonic power is proportional to (*Q*/*V*)^2^, when phase-matching is satisfied, the small volume of the tightly confined modes will result in improved conversion efficiencies.

### Second Harmonic Generation

**Figure 2:**
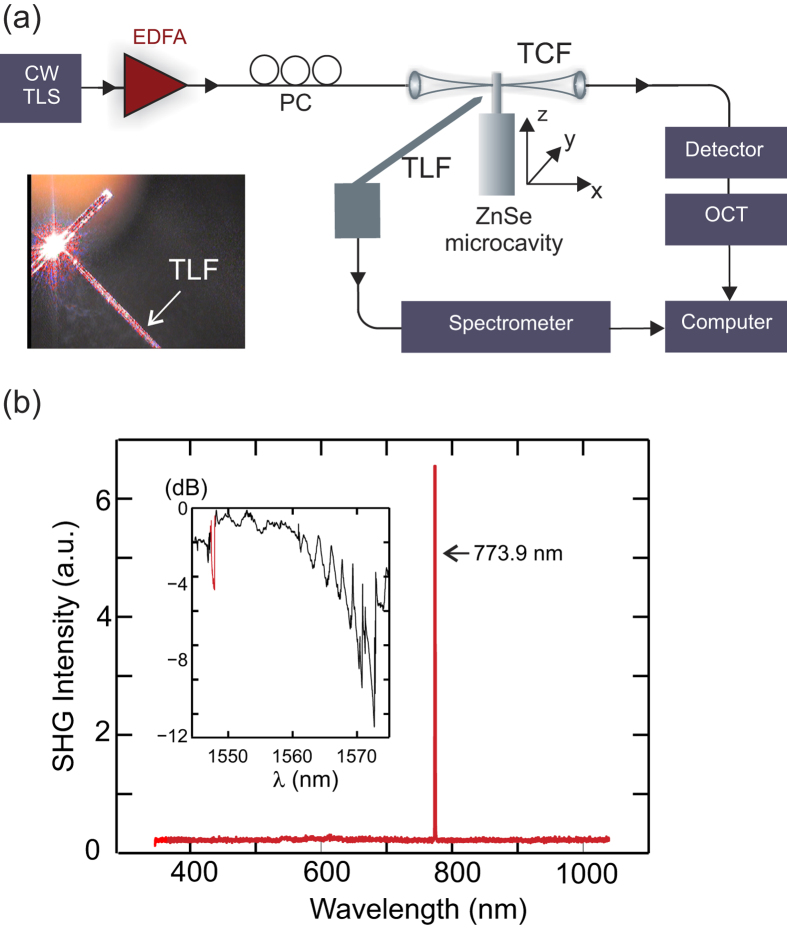
Second harmonic generation. (**a**) Experimental set-up including a tunable laser source (TLS), ebrium doped fibre amplifier (EDFA), polarization control (PC), tapered coupling fibre (TCF), optical components tester (OCT), and a tapered lensed fibre (TLF). Inset shows a photograph of the generated visible second harmonic light. (**b**) Measured emission spectrum at 773.9 nm; inset displays the corresponding transmission spectrum showing the pump resonance at *λ* ~ 1548 nm, with a *Q* ~ 4.1 × 10^3^ (red fit).

To investigate SHG in the ZnSe resonators we employed the experimental set-up shown in Fig. 2(a). The resonator was pumped with a standard telecommunications band tunable continuous wave source, that was amplified via an erbium doped fibre amplifier (EDFA) to boost the power before coupling into the resonator using a silica tapered coupling fibre (TCF) with a waist diameter of 1–2 *μ*m. In order to observe the second harmonic light, the coupling conditions had to be precisely tuned to locate a resonance close to phase-matching. As well as controlling the polarization of the coupled modes, we found that it was also necessary to slightly tune the angle of the taper with respect to the resonator (by an amount no larger than 2°). As will be discussed below, adjusting the coupling angle essentially allowed us to modify the height of the mode circulating in the cylinder, thus providing an additional degree of freedom to obtain phase-matching over that of the micro-disks. As the second harmonic associated with the telecommunications band pump falls on the red edge of the visible spectrum, it is straightforward to detect when phase-matching has been achieved by imaging the resonator on a CCD camera, as illustrated by the photograph in the inset of Fig. 2(a). To measure the spectral content of the generated light a tapered lensed fibre (TLF) was positioned in close proximity (as labelled). This was necessary as the short wavelength harmonic, which must be in a higher order mode to satisfy phase-matching, did not have sufficient overlap (<1% of the mode energy^23^) with the micron-sized fibre used to couple the pump. A typical emission spectrum obtained for the SHG process is shown in Fig. 2(b), with the transmission spectrum measured for the coupling conditions displayed in the inset. We note that the coupled resonance has a reduced *Q*_*l*_ ~ 10^3^, which broadens the resonance to ~0.2 nm. Accounting for this, the position of the generated light is in excellent agreement with the resonance at 1548 nm, i.e., *λ*_*S*_ = 1548/2 ~ 773.9 nm. Furthermore, no other emission lines were observed within the range of the spectrometers (350–1700 nm), indicating that SHG was the dominant nonlinear process.

**Figure 3:**
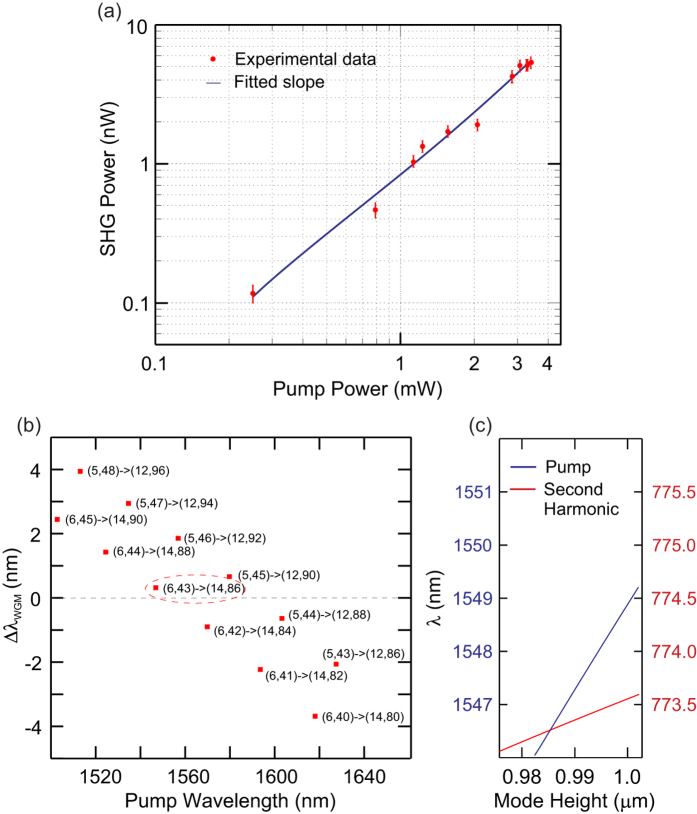
Harmonic power scaling and phase-matching. (**a**) Measured second harmonic power collected in the TLF as a function of the fundamental power dropped into the resonator. (**b**) Calculated difference between the resonance positions (Δ*λ*_WGM_) for mode pairs in our microcylindrical resonators near phase-matching. (**c**) Tuning the mode height to obtain Δ*λ*_WGM_ = 0; blue curve is position of pump resonance and red is for the second harmonic resonance.

To confirm the second harmonic nature of the process, Fig. 3(a) shows a logarithmic plot of the collected second harmonic power (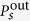) as a function of the power dropped into the resonator (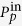), where it can be seen that the minimum coupled power at which we recorded a measurable harmonic signal was 250 *μ*W. Fitting the data with a linear curve we obtain a slope of 1.95 ± 0.10, which is in good agreement with the expected quadratic dependence. Using this value we can then extract the external efficiency as 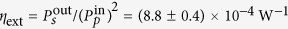. Significantly, this external efficiency is comparable to what has been obtained via a similar modal phase-matching method in a smaller, 3.8 *μ*m diameter microresonator fabricated from AlGaAs^15^ (a material with a larger second order nonlinearity^24^); a good indication that the overall efficiency of our system is substantial. It is also worth noting that in contrast to previous reports in GaAs-based micro-disks^14^,^15^, our measurements do not show any evidence of a roll-off in second harmonic power associated with thermal shifting of the pump resonance. We attribute this behaviour, or lack of, to the order of magnitude lower thermo-optic coefficient of the ZnSe material compared to GaAs, which makes it more suitable for high power pumping.

To better understand the phase-matching considerations in our resonators we have used a semi-analytic approach to the determine the WGMs, and their corresponding radial and azimuthal (n,l) mode numbers, for both the pump and second harmonic wavelengths (see Methods). To satisfy phase-matching in the cylindrical geometry it is necessary to find pairs of modes that not only conserve energy *λ*_*p*_ = 2*λ*_*s*_, but also the azimuthal component of the orbital momentum *l*_*s*_ = 2*l*_*p*_^10^. Figure 3(b) plots Δ*λ*_WGM_ = *λ*_WGM*p*_ − 2*λ*_WGM*s*_ (i.e., the difference in resonance positions for the pump and signal) for a selection of modes in the vicinity of Δ*λ*_WGM_ ≈ 0, calculated assuming TE polarization and a mode height of ~1 *μ*m (see Methods). As it can be seen, none of the mode pairs precisely satisfy the phase-matching condition, although the combination of (n_*p*_,l_*p*_) = (6,43) and (n_*s*_,l_*s*_) = (14,86) is very close with Δ*λ*_WGM_ ~ 0.3 nm. However, a convenient way to fine tune the resonance positions within the cylindrical geometry is to adjust the mode height, which can be achieved by introducing a slight angle into the coupling taper. The effect of angle coupling typically acts to reduce the mode height as the beam size in a cylindrical resonator is inversely proportional to the divergence angle^25^. This phase-matching method is illustrated in Fig. 3(c) which shows that for a slight decrease in the mode height to 0.98 *μ*m, it is possible to shift the positions of the resonance pair (6,43) and (14,86) to obtain perfect phase-matching and efficient generation of the second harmonic as in Fig. 2(b). Importantly, this approach can also be applied to achieve phase-matching with other Δ*λ*_WGM_ ≈ 0 pairs plotted in Fig. 3(b), and similarly for the TM modes (not shown here), as we discuss below.

### Tuning the frequency conversion

**Figure 4:**
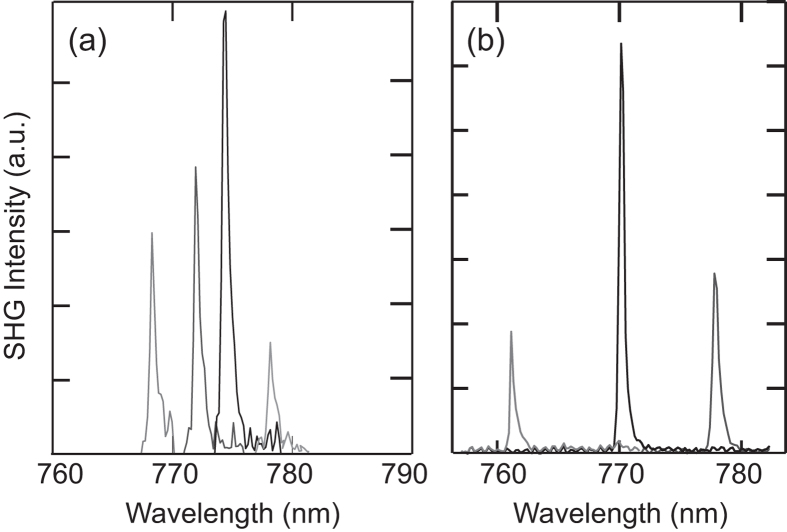
Tunable frequency conversion. Measured second harmonic emission (relative counts) for different pump wavelengths in ZnSe resonators with diameters of (**a**) 15 *μ*m and (**b**) 20 *μ*m.

To demonstrate the value of this additional degree of control over the phase-matching conditions, Fig. 4(a) shows how the second harmonic emission can be tuned over 10 nm, corresponding to 20 nm of pump tuning in Fig. 3(b). To achieve SHG over this range the coupling was optimized through a combination of adjusting the polarization to tune between the TE and TM modes, and the angle of the TFC to vary the mode height. It is clear from these spectra that although the maximum second harmonic efficiency is obtained for the original mode pair with the smallest Δ*λ*_WGM_, it is nevertheless possible to phase-match several different mode pairs: *λ*_*P*1_ = 1537 nm and *λ*_*s*1_ = 768.3 nm, *λ*_*P*2_ = 1544 nm and *λ*_*s*2_ = 772 nm, *λ*_*P*3_ = 1548.8 nm and *λ*_*s*3_ = 774.4 nm, *λ*_*P*4_ = 1556.4 nm and *λ*_*s*4_ = 778.2 nm . Significantly, we believe that this is the largest tunability of harmonic generation demonstrated in a resonator of fixed size^26^. However, as we have fabricated ZnSe fibres with other core diameters, these provide a convenient means through which to investigate tuning the SHG even further.

Although the majority of our work was focused on the 15 *μ*m diameter resonators owing to their favourable dimensions for phase-matching SHG in the window of our EDFA, resonators with diameters spanning 10–150 *μ*m were also investigated and found to support WGMs. Figure 4(b) shows SHG emission that was obtained when using a resonator fabricated from a 20 *μ*m ZnSe core fibre. For this larger diameter fibre, the coupled mode analysis predicts that optimal phase-matching should in fact occur at a longer pump wavelength of ~2 *μ*m. However, the increase in diameter results in a substantial decrease of the FSR to ~18 nm, almost half of that of the 15 *μ*m resonator, thus increasing the density of modes that are available for phase-matching. As a result, the second harmonic emission can now be tuned across a ~20 nm span, corresponding to the pump and second harmonic pairs: *λ*_*p*1_ = 1523 nm and *λ*_*s*1_ = 761 nm, *λ*_*p*2_ = 1541 nm and *λ*_*s*2_ = 770 nm, *λ*_*p*3_ = 1556 nm and *λ*_*s*3_ = 778 nm. We note that the observed second harmonic tuning shown here is primarily limited by the bandwidth of the EDFA used to boost the pump light, and our modelling predicts that it should be possible to phase-match selected pairs in this resonator over several hundreds of nanometers, and up to *λ*_*p*_ = 2*μ*m at least.

## Discussion

We have presented the first demonstration of intermodal phased-matching of second harmonic generation in a ZnSe resonator. This observation has been enabled by our unique fibre-based fabrication method that allows for the formation of crystalline microcylindrical resonators with ultrasmooth surfaces. By adjusting the coupling conditions we have demonstrated tunability of the SHG over 20 nm, significantly larger that what has been reported in other fixed diameter resonators^26^. In this regard, a potential route to obtaining even broader band operation would be to taper the ZnSe fibres using standard processing methods, so that the core diameter varies continuously along the length^27^. With this approach, it would then be possible to realize optimal phase-matching of different pump wavelengths by simply scanning the tapered coupling fibre along the resonator axis.

Using our current coupling scheme we have measured a conversion efficiency of 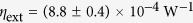. However, we expect that there is considerable scope to optimize this further as the high refractive index contrast between the silica coupler and the ZnSe resonator results in coupling to higher order radial modes, as illustrated in Fig. 3(b). Although in future work we will look to address this by using higher index couplers (e.g., a prism or chalcogenide tapered fibre) to excite modes with an improved overlap^10^, the tapered silica fibre approach is convenient as it facilitates integration with conventional fibre infrastructures. Furthermore, the combination of the small mode volume and the high nonlinearity of the ZnSe material means that the frequency conversion in our system can still be achieved at ultra-low power levels, so that we can expect these *χ*^(2)^ resonators to find use in wide ranging applications in both the classical and quantum optical domains. Importantly, owing to the ease at which the second harmonic emission can be tuned across the various resonance conditions, it should also be possible to phase-match more complex processes such as sum and difference-frequency generation^4^,^26^, eventually allowing for wavelength conversion across the full 500–22000 nm transmission window.

## Methods

### ZnSe resonator fabrication

The ZnSe core fibres were fabricated by depositing the semiconductor material inside silica capillaries with inner diameters of 15 *μ*m and 20 *μ*m using a high pressure microfluidic chemical technique^18^. In this process, a mixture of dimethylzinc and dimethylselenide precursors (SAFC Hitech) were configured to flow through the capillaries together with a hydrogen carrier gas. The partial pressures for the organometallic precursors were ~20–40 kPa, with the carrier around 70 MPa, and their molar ratio was optimized to achieve the desired stoichiometry, morphology and material quality. The deposition was conducted at a temperature of 450 °C over 24 hours. For this particular precursor chemistry, deposition of the material stops before complete filling of the capillary as the reaction byproduct (methane) must be exhausted through an interior channel. However, this channel can be as small as a few hundred nanometers and thus has little impact on the resonator modes that are largely confined to the smooth outer surface. Following the fibre fabrication, the microcylindrical resonators were formed by etching the silica cladding away from the ZnSe core using a buffered hydrofluoric acid (HF) solution (volume ratio 20:1 of 40% ammonium fluoride in water and 49% HF in water), before rinsing with deionized water.

### Resonator characterization

A single tunable external cavity CW laser source (Tunics Plus: tuning range of 1490–1620 nm and linewidth of ~400 kHz) was coupled to the ZnSe resonator using a tapered single mode silica fibre with a waist diameter of 1–2 *μ*m (see Fig. 2(a)). Micro-positioning stages were used to place the resonator in close proximity to the taper and optimize coupling to the phase-matched modes. A polarization controller was positioned before the taper to selectively couple into either the TE or TM modes of the cylindrical resonator. The spectral output from the taper was monitored using an optical component tester (Yenista CT400) with a 1 pm resolution. For the SHG measurements, we modified the set-up so that the CW source was amplified by a Keopsys C-band low noise amplifier. This amplifier has a specified operating range of 1529–1562 nm, though note that the tail of the gain curve extends down to ~1520 nm.

### Modelling

The optical modes of a cylindrical resonator can be calculated by solving the Helmholtz equation in cylindrical coordinates^28^. We search for modes around the fundamental pump (~1550 nm) and the second harmonic (~775 nm) wavelength regions, using the material dispersion equation in Ref. 29. Our analysis is restricted to search for modes that have heights on the order of 1–2 *μ*m, as this range has previously been found to provide good agreement with the experimental findings in the high index semiconductor material resonators^17^. In particular, we have found that the set of modes which best match the experimental conditions in Fig. 2 is obtained for a mode height of ~1 *μ*m.

## Additional Information

**How to cite this article**: Vukovic, N. *et al.* Tunable continuous wave emission via phase-matched second harmonic generation in a ZnSe microcylindrical resonator. *Sci. Rep.* **5**, 11798; doi: 10.1038/srep11798 (2015).
