# Corrigendum: Tunable continuous wave emission via phase-matched second harmonic generation in a ZnSe microcylindrical resonator

**DOI:** 10.1038/srep22825

**Published:** 2016-03-17

**Authors:** N. Vukovic, N. Healy, J. R. Sparks, J. V. Badding, P. Horak, A. C. Peacock

Scientific Reports 5: Article number: 11798; 10.1038/srep11798 published online: 07022015; updated: 03172016

The Acknowledgements section in this Article is incomplete.

“The authors acknowledge EPSRC (EP/J004863/1 and EP/I035307/1), NSF (DMR-1107894), the Penn State Materials Research Science and Engineering Center (NSF DMR-0820404), and the Air Force Research Laboratory (FA8650-10-C-1902) for financial support. The data for this work is accessible through the University of Southampton Institutional Research Repository (doi: 10.5258/SOTON/376815).”

should read:

“The authors acknowledge EPSRC (EP/J004863/1 and EP/I035307/1), NSF (DMR-1107894), the Penn State Materials Research Science and Engineering Center (NSF DMR-0820404), and the Air Force Research Laboratory (FA8650-10-C-1902) for financial support. The data for this work is accessible through the University of Southampton Institutional Research Repository (doi: 10.5258/SOTON/376815). This work was also supported by the Center for Nanoscale Science, an NSF Materials Research Science and Engineering Center, under the award DMR-1420620 to JVB.”

